# Co-expression of PKM2 and TRIM35 predicts survival and recurrence in hepatocellular carcinoma

**DOI:** 10.18632/oncotarget.2991

**Published:** 2014-12-11

**Authors:** Zhiao Chen, Xinyuan Lu, Zhichao Wang, Guangzhi Jin, Qifeng Wang, Di Chen, Taoyang Chen, Jinjun Li, Jia Fan, Wenming Cong, Qiang Gao, Xianghuo He

**Affiliations:** ^1^ Fudan University Shanghai Cancer Center and Institutes of Biomedical Sciences, Department of Oncology, Shanghai Medical College, Fudan University, Shanghai, China; ^2^ Department of Pathology, Eastern Hepatobiliary Surgery Hospital, Second Military Medical University, Shanghai, China; ^3^ Liver Cancer Institute, Zhongshan Hospital, and Key Laboratory of Carcinogenesis and Cancer Invasion (Ministry of Education), Fudan University, Shanghai, China; ^4^ Department of Pathology, Fudan University Shanghai Cancer Center, Shanghai, China; ^5^ State Key Laboratory of Oncogenes and Related Genes, Shanghai Cancer Institute, Renji Hospital, Shanghai Jiao Tong University School of Medicine, Shanghai, China; ^6^ Qi Dong Liver Cancer Institute, Qi Dong, Jiangsu, China

**Keywords:** Pyruvate kinase isoform M2, Tripartite motif-containing protein 35, Hepatocellular carcinoma, Prognosis, biomarker

## Abstract

The identification of prognostic markers for hepatocellular carcinoma (HCC) is needed for clinical practice. Tripartite motif-containing 35 (TRIM35) is a tumor suppressor of HCC. TRIM35 inhibits phosphorylation of pyruvate kinase isoform M2 (PKM2), which is involved in aerobic glycolysis of cancer cells. We found that expression of PKM2 was significantly increased in HCC tissues. This overexpression of PKM2 was correlated with a high TNM stage and level of vascular invasion. Patients with HCC who were positive for PKM2 expression and negative for TRIM35 expression had shorter overall survival and time to recurrence than patients who were negative for PKM2 and positive for TRIM35. Furthermore, PKM2/TRIM35 combination was an independent and significant risk factor for recurrence and survival. In conclusion, PKM2 (+) and TRIM35 (−) contribute to the aggressiveness and poor prognosis of HCC. PKM2/TRIM35 expression could be a biomarker for the prognosis of HCC and target for cancer therapy.

## INTRODUCTION

Hepatocellular carcinoma (HCC) is one of the most common malignant tumors worldwide and is the third leading cause of cancer-related deaths. Further, HCC incidence and mortality are especially high in China [1, 2]. To date, hepatic resection and liver transplantation have remained the most effective treatment with curative potential. However, the 5-year survival rate is only about 60% in well-selected patients [3]. One of the reasons for the poor prognosis of HCC is the high rate of recurrence due to intrahepatic metastasis or the multicentric development of each neoplastic clone, which can be attributed to the lack of reliable markers for tumor recurrence and metastasis [4].

A review of the evidence has supported the general hypothesis that cancer is primarily a disease of energy metabolism [5]. With recent advances in research on tumorigenesis, the importance of a unique metabolic phenotype in tumors is gradually becoming understood. Considerable attention has been paid to the roles of aerobic glycolysis (Warburg effect) in cancer cells [6, 7]. Pyruvate kinase (PK) catalyzes a crucial step in glycolysis. PKM2 (but not the PKM1 isoform) promotes this pathway in cancer cells [8], thus facilitating the switch from oxidative phosphorylation to aerobic glycolysis [9, 10]. In recent years, PKM2 up-regulation has been observed in numerous cancers, including lung [11], gastric [12], cervical [13], colorectal cancers [14], and hepatocellular carcinoma [15]. However, no study has previously been performed to assess i) whether there are any correlations among the protein level of PKM2, clinicopathological parameters, and the survival rate of HCC and ii) to examine if PKM2 can be used as a potential biomarker and target for HCC therapy.

In a previous study, we identified tripartite motif-containing 35 (TRIM35) as a novel tumor suppressor of hepatic carcinogenesis [16]. TRIM35 inhibits the Warburg effect and suppresses the tumorigenicity of HCC cells through the blockade of PKM2 Y105 phosphorylation [17]. In the current study, we investigated the expression levels of PKM2 and TRIM35 in HCC and paracancerous tissues, as well as their relationships with clinicopathological parameters and prognosis. The aim of this study was first, to identify the influences of PKM2 and TRIM35 expression on tumor aggressiveness in patients with HCC, and second, to validate these risk factors in patients with early recurrence.

## RESULTS

### Baseline characteristics and clinical outcomes of the patients in this study

We obtained snap-frozen or formalin-fixed paraffin-embedded (FFPE) HCC tissues and adjacent non-tumor liver tissues from 688 patients with HCC who had undergone curative resection between 2000 and 2013. *PKM2* and *TRIM35* mRNA levels were determined in cohort 1 using a quantitative RT-PCR assay because only RNA samples were available. In cohorts 2, 3 and 4, PKM2 and TRIM35 levels were determined using immunohistochemistry tissue microarrays. For the primary group, archived tissue samples for the tissue microarray construction were obtained from patients who received curative resection of HCC between January and December 2007. The median follow-up period was 60.0 months (range, 3.0-74.0; SD, 25.3) and the postoperative cumulative survival and recurrence rates (in parentheses) at 1, 3, and 5 years were 84.2% (72.7%), 68% (62.4%), and 66.4% (53.5%), respectively. For the validation group, FFPE tissues of HCC nodules were collected from patients between January and December 2000. The median follow-up period was 29.0 months (range, 1.0-141.0; SD, 43.1) and the postoperative cumulative survival and recurrence rates (in parentheses) at 1, 3, and 5 years were 62% (55%), 45% (41%), and 22% (18%), respectively. Patients did not have signs of distant metastasis, nor had they received anticancer therapy before surgery. Cohort 4 included 118 patients with HCC who had first undergone radical resection of HCC, had a relapse a few years later, and then underwent a second resection of HCC.

Most of the HCC patients in the four cohorts were men (85.5%), were carriers of hepatitis B virus (HBV) (82.6%), had liver cirrhosis (72.8%), had an elevated serum alpha-fetoprotein (AFP) level (61.7%), and had a single tumor nodule at the time of resection (83.7%) (Supplementary Table 1). Clinical variables were similar in the four patient cohorts, with the exception of hepatitis history, liver cirrhosis, tumor size, tumor number, and vascular invasion. As compared with the patients in the other cohorts, fewer patients were HBV carriers in cohort 1; fewer patients in cohort 1 and more patients in cohorts 2 and 3 had liver cirrhosis; and more patients in cohort 4 had small tumors. Moreover, most of the patients in cohort 3 had vascular invasion.

### PKM2 is significantly increased in HCC

**Figure 1:**
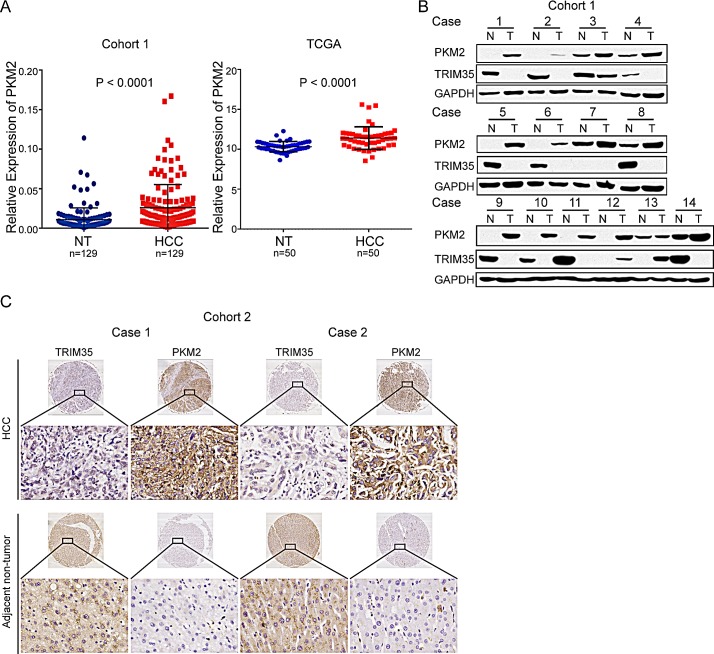
PKM2 is significantly increased in HCC (A) The expression levels of PKM2 were measured by quantitative real-time PCR in 129 tumor and adjacent non-tumor liver tissues (left). The expression levels of PKM2 in the TCGA are also presented (right). Data are depicted as log_2_ of the RPKM (read per kilo bases per million reads). (B) Protein levels of PKM2 and TRIM35 in 14 representative HCC tissues (T) and adjacent non-tumor liver tissues (N) were analyzed by western blotting. (C) Two representative cases of PKM2 and TRIM35 expression are shown for HCC tissues and adjacent non-tumor liver tissues. Original magnification, ×400.

In the previous study, we applied gene expression profiling to 49 HCCs and matched adjacent non-tumor liver tissues [16]. Our results showed that PKM2 was significantly increased in HCC tissues (Supplementary Figure 1). In the present study, we confirmed that PKM2 expression was significantly increased in the HCC tissues of the patients in cohort 1 and in The Cancer Genome Atlas (TCGA) database, as detected by quantitative real-time PCR or a microarray for its mRNA level (Figure 1A). Furthermore, we used immunoblotting to examine the expressions of PKM2 and TRIM35 in 14 paired tumorous liver tissues and adjacent non-tumorous liver tissues from cohort 1. The results showed that tumorous liver tissues exhibited increased PKM2 expression and the loss of or substantial decreases in TRIM35 expression, as compared with the non-tumorous liver tissues (Figure 1B). We also performed a tissue array to analyze the protein levels of TRIM35 and PKM2 using immunohistochemical staining in 236 HCC tissues, as compared with the levels in matched adjacent non-tumor liver tissues. The results showed that TRIM35 and PKM2 were primarily localized to the cytoplasm (Figure 1C). Positive PKM2 expression was found in 77 of the 236 (32.6%) primary HCC samples and none of the adjacent non-tumor tissues (P < 0.001), whereas positive TRIM35 expression was found in 159 of the 236 (67.4%) primary HCC samples and all of the adjacent non-tumor tissues (P < 0.001), indicating that increased PKM2 expression and decreased TRIM35 expression are frequent events in HCC.

### Positive expression of PKM2 and negative expression of TRIM35 significantly correlates with cancer progression and poor prognosis in HCC patients

As the HCCs had shown increased PKM2 expression and decreased TRIM35 expression, we performed further analyses to determine the clinicopathological significance of PKM2 and TRIM35 in HCC. The expression level of TRIM35 was negatively correlated with the tumor size, histological grade, and AFP concentration (data not shown). These findings are consistent with our previous observations [16]. Importantly, we also found that PKM2 overexpression was correlated with serum AFP concentration, tumor size, microvascular invasion, poor differentiation, and higher TNM stage (Table 1).

**Table 1:** Relationship of PKM2 expression level with the clinicopathological features in HCC

Characteristics	Primary group			Validation group		
	Negative	Positive	*P*	Negative	Positive	*P*
Age, years						
≤ 51	71	50	0.004	30	69	0.483
> 51	88	27		37	69	
Gender						
Female	18	12	0.406	10	23	0.750
Male	141	65		57	115	
Hepatitis history						
No	1	2	0.249	17	30	0.599
Yes	158	75		50	106	
α-Fetoprotein (ng/ml)						
≤ 20	70	23	0.046	28	47	0.281
> 20	89	54		39	91	
Liver cirrhosis						
No	17	11	0.520	7	14	0.947
Yes	142	66		60	124	
Tumor size (cm)						
≤ 5	82	28	0.037	26	54	0.964
> 5	77	49		41	84	
Tumor number						
Single	129	65	0.590	47	115	**0.030**
Multiple	30	12		20	23	
Vascular invasion						
No	116	36	<0.0001	33	33	**<0.001**
Yes	43	41		34	105	
Tumor differentiation						
I-II	124	39	<0.0001	15	19	**0.120**
III-IV	35	38		52	119	
TNM stage						
I	100	29	0.001	27	29	**<0.001**
II	35	23		25	95	
III	24	25		15	14	

Statistical analyses were done by the Chi-square (χ2) test.

**Figure 2:**
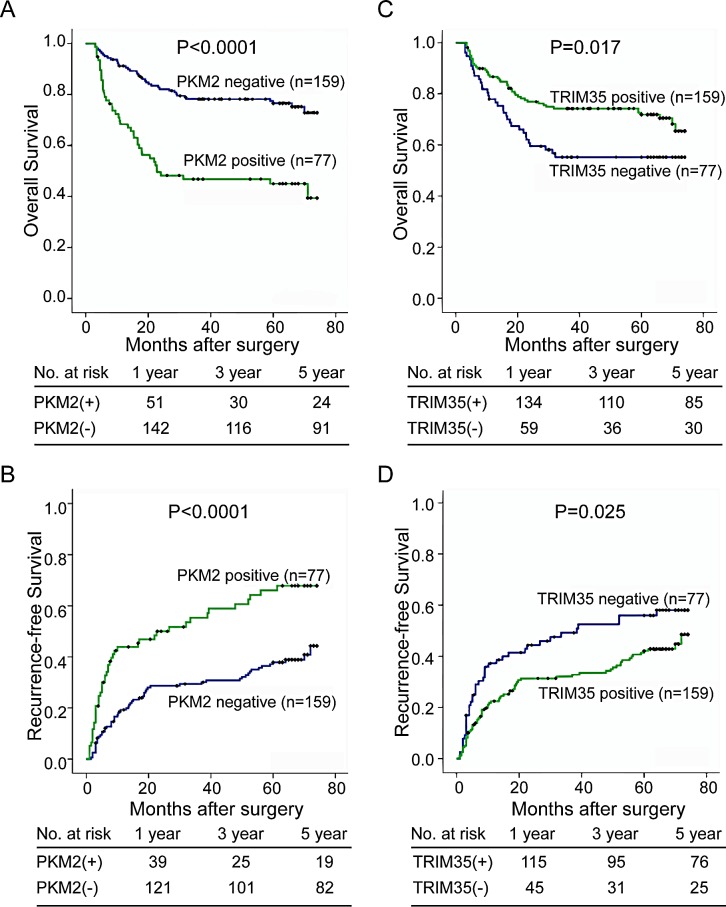
Positive expression of PKM2 and negative expression of TRIM35 significantly correlates with poor prognosis in HCC patients (A, B) Kaplan-Meier analysis of the correlation between PKM2 expression and the recurrence-free or overall survival of 236 patients with HCC. Log-rank tests were used to determine statistical significance. (C, D) Kaplan-Meier analysis of the correlation between TRIM35 expression and the recurrence-free or overall survival. Log-rank tests were used to determine statistical significance.

In addition, we evaluated the clinical relevance of PKM2 and TRIM35 expression to prognosis in the primary cohort. At the time of last follow-up, 135 and 121 of the 236 patients had tumor recurrence and had died, respectively. Kaplan–Meier analysis showed that the median overall survival (OS) time was 40.3 (95% CI: 33.3–47.4) months for patients with HCC who were positive for PKM2 and 60.8 (95% CI: 56.9–64.6) months for patients with HCC who were negative for PKM2 (P < 0.0001, log rank test, Figure 2A). The median time to recurrence (TTR) was 34.8 (95% CI: 27.5–42.0) months for patients with HCC who were positive for PKM2 and 52.9 (95% CI: 48.4–57.5) months for patients with HCC who were negative for PKM2 (P < 0.0001, log-rank test, Figure 2B). Furthermore, we found that patients with negative TRIM35 expression had shorter OS (47.0 versus 57.7 months, P = 0.017, log-rank test, Figure 2C) and TTR (40.0 versus 50.7 moths, P = 0.025, log-rank test, Figure 2D).

### Combined influence of PKM2 and TRIM35 dimorphisms on risk of HCC death and recurrence

**Figure 3:**
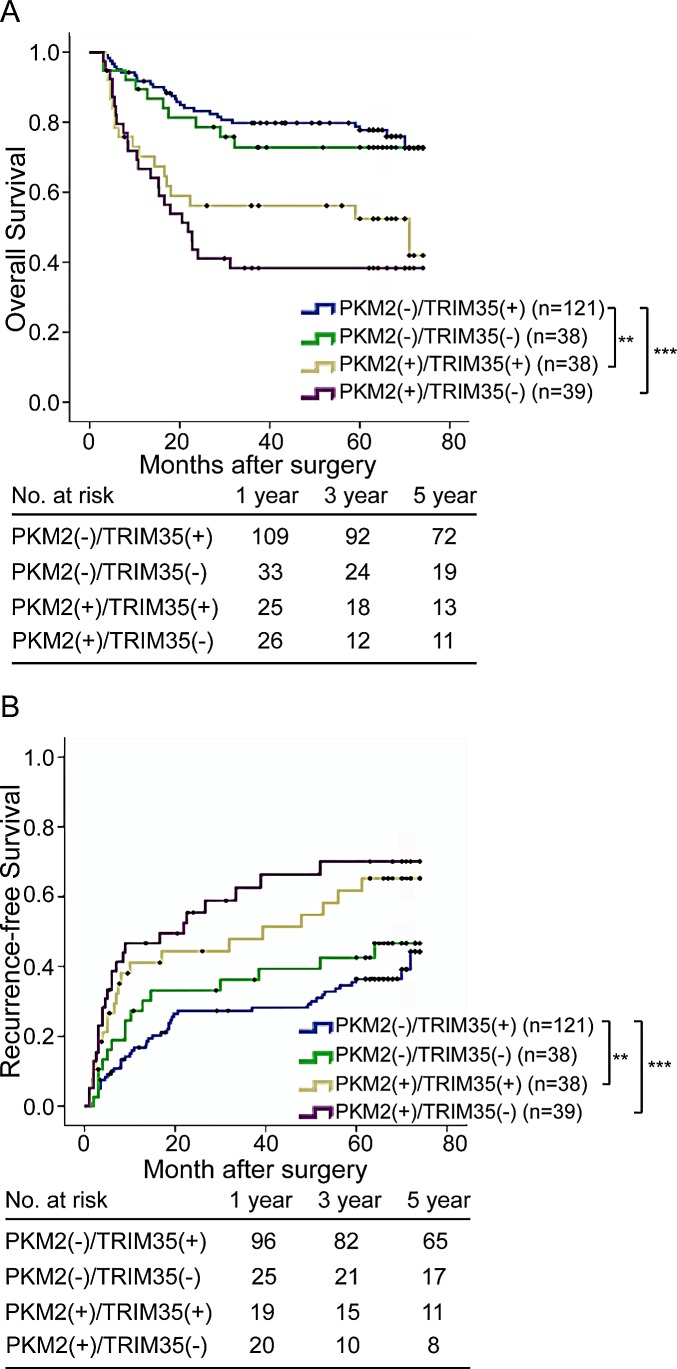
Combined influence of PKM2 and TRIM35 dimorphisms on risk of HCC death and recurrence The associations of PKM2/TRIM35 co-expression with recurrence and overall survival in patients with HCC. **P < 0.001; ***P < 0.0001.

After identifying the risks associated with PKM2 and TRIM35 expression, we classified the 236 HCC patients in the primary cohort into 4 groups according to the expression levels of PKM2 and TRIM35: group I patients were only positive for TRIM35, group II patients were negative for both markers, group III patients were positive for both markers, and group IV were only positive for PKM2. Using the Kaplan–Meier method, patients with the PKM2 (+)/TRIM35 (−) expression had the shortest TTR (median months: 31.4, 95% CI: 21.5–41.4) and OS (median months: 36.2, 95% CI: 26.7–45.8), whereas patients with the PKM2 (−)/TRIM35 (+) expression pattern had the longest TTR (median months: 54.3, 95% CI: 49.2–59.5) and OS (median months: 61.6, 95% CI: 57.3–65.8) (Figure 3).

In an attempt to investigate the value of PKM2/TRIM35 expression for HCC prediction, the validation cohort was used to demonstrate the influence of PKM2/TRIM35 expression on patient prognosis and outcome. In the validation cohort, positive PKM2 expression was found in 138 of 205 (67.3%) primary HCC samples, whereas positive TRIM35 expression was found in 149 of 205 (72.7%) primary HCC samples. PKM2 overexpression was correlated with tumor number, microvascular invasion and higher TNM stage (Table 1). Furthermore, patients with HCC who were positive for PKM2 expression and negative for TRIM35 expression had shorter OS and TTR than patients with HCC who were negative for PKM2 expression and positive for TRIM35 expression (Supplementary Figure 2). In addition, as in the primary cohort, PKM2 combined with TRIM35 expression (Supplementary Figure 3) was found to be significantly associated with both OS and TTR in the validation cohort.

### Multivariate analyses and HCC risk prediction model

To test whether the expression levels of PKM2 and TRIM35 were independent of other predictive variables, we applied univariate and multivariate analyses using a Cox multivariate proportional hazard regression model with PKM2/TRIM35 expression and clinicopathologic factors (such as age, gender, tumor size, vascular invasion, and stage) as covariates. Univariate analysis showed that vascular invasion, TNM stage, the expressions of PKM2 and TRIM35, and the PKM2/TRIM35 combination were the factors that were consistently significant for both OS and TTR in the primary and validation cohorts (Table 2 and Supplementary Table 2). Serum AFP concentration and tumor size were significant factors for the primary cohort, but not for the validation cohort. Tumor multiplicity was found to be predictive of OS and TTR in the validation cohort, but not in the primary cohort.

**Table 2:** Univariate analyses of factors associated with OS and TTR in primary HCC cohort

Factors	OS Relative risk	(95% CI)	P Value	TTR Relative risk	(95% CI)	P Value
Age, years (>51 vs.≤51)	0.977	0.630-1.515	0.977	1.082	0.740-1.581	0.686
Gender (male vs. female)	1.451	0.699-3.013	0.318	0.964	0.559-1.665	0.896
Hepatitis history (yes vs. no)	0.377	0.092-1.535	0.377	0.477	0.118-1.935	0.300
α-Fetoprotein (ng/ml) (>20 vs. ≤20)	2.199	1.336-3.620	**0.002**	1.592	1.066-2.380	**0.023**
Tumor differentiation (poor vs. well)	2.643	1.703-4.103	**0.000**	1.471	0.987-2.193	0.058
Tumor size (cm) (>5 vs. ≤5)	3.089	1.888-5.054	**0.000**	2.134	1.438-3.166	**0.000**
Tumor multiplicity (multiple vs. single )	1.237	0.715-2.141	0.446	1.509	0.957-2.379	0.076
Vascular invasion (yes vs. no)	4.401	2.790-6.944	**0.000**	2.629	1.789-3.864	**0.000**
TNM stage (III vs. II vs. I)	2.256	1.809-3.067	**0.000**	1.940	1.541-2.442	**0.000**
PKM2 (Pos vs. Neg)	3.062	1.971-4.756	**0.000**	2.306	1.570-3.387	**0.000**
TRIM35 (Neg vs. Pos)	0.586	0.376-0.913	**0.018**	0.643	0.435-0.950	**0.027**
Combination of PKM2 and TRIM35						
Overall	1.576	1.320-1.883	**0.000**	1.416	1.212-1.655	**0.000**
II vs. I	1.194	0.580-2.459	0.630	1.316	0.742-2.333	0.348
III vs. I	1.645	1.222-2.213	**0.001**	1.506	1.165-1.948	**0.002**
IV vs. I	1.552	1.292-1.863	**0.000**	1.395	1.183-1.646	**0.000**

Univariate analysis was calculated by the Cox proportional hazards regression model. Group I patients were only positive for TRIM35, group II patients were negative for both markers, group III patients were positive for both markers, and group IV were only positive for PKM2. TNM, tumor-nodes-metastases; CI, confidential interval.

In addition, a multivariate statistical analysis revealed that HCC patients with the signature of TRIM35 (−)/PKM2 (+) expression harbored a 2.25-fold higher risk of cancer recurrence (95% CI: 1.37–3.71, P < 0.001) and a 2.1-fold higher risk of death (95% CI: 1.19–3.71, P < 0.001) in the primary cohort (Table 3). Thus, this pattern had an increased prognostic value compared with TRIM35 or PKM2 alone. Importantly, we validated these identified risk factors in cohort 3. Cox proportional hazards regression analysis confirmed that TRIM35 (−)/PKM2 (+) expression was a significant risk factor for OS and TTR in the validation cohort (for OS: hazard rate [HR] = 2.67, 95% CI, 1.63–4.35, P < 0.001; for TTR: HR = 2.33, 95% CI, 1.43–3.80, P = 0.001) (Table 3). These results indicate that the signature of TRIM35/PKM2 expression is an independent and significant factor for reduced TTR and OS.

**Table 3:** Independent prognostic factors for OS and TTR by multivariate analyses in primary and validation cohort

	Variables	Primary cohort			Validation cohort		
		HR	95%CI	*P*	HR	95%CI	*P*
OS	Tumor differentiation (poor vs. well)	1.69	1.06-2.69	**0.027**			NS
	Vascular invasion (yes vs. no)	1.86	1.10-3.14	**0.020**			NS
	TNM stage (III vs. II vs. I)			NS	1.62	1.20-2.20	**0.002**
	PKM2 (Pos vs. Neg)	2.22	1.41-3.50	**0.001**	1.72	1.14-2.59	**0.009**
	Combination of PKM2 and TRIM35						
	Overall			**0.007**			**<0.001**
IV vs. I	2.10	1.19-3.71	**0.011**	2.67	1.63-4.35	**<0.001**
TTR	TNM stage (III vs. II vs. I)	1.39	1.05-1.85	**0.023**	1.55	1.14-2.10	**0.005**
	TRIM35 (Neg vs. Pos)			NS	0.50	0.33-0.75	**0.001**
	PKM2 (Pos vs. Neg)	1.96	1.32-2.90	**0.001**	1.56	1.04-2.34	**0.030**
	Combination of PKM2 and TRIM35						
	Overall			**0.007**			**0.001**
	IV vs. I	2.25	1.37-3.71	**0.001**	2.33	1.43-3.80	**0.001**

Multivariate analysis was performed using the Cox multivariate proportional hazard regression model with stepwise manner (forward, likelihood ratio). Group I patients were only positive for TRIM35, group II patients were negative for both markers, group III patients were positive for both markers, and group IV were only positive for PKM2. TNM, tumor-nodes-metastases; HR, hazard ratio; CI, confidential interval; NS, not statistically significant.

To enhance the diagnostic value of PKM2/TRIM35 expression for early recurrence of HCC, we further performed a receiver-operating characteristic (ROC) curve analysis using the IHC data from cohort 4 to determine the efficacy of PKM2 and TRIM35 expression in discriminating early recurrence (less than 3 years) in patients with HCC. As shown in Supplementary Figure 4, the area under the curve (AUC) was 0.640 for PKM2 and 0.600 for TRIM35. Although the AUC for PKM2 was significantly different from random assignment (AUC = 0.5), the AUC was less than 0.7, which indicated that PKM2 expression level had low efficacy. However, AUC was 0.704 for the PKM2/TRIM35 combination, suggesting that the AUC for the combined marker was higher than that for the individual marker.

## DISCUSSION

Although there have been tremendous clinical achievements in treatment for HCC during the past few decades, the prognosis of patients with HCC is still unsatisfactory. Improving the survival rate of patients with HCC requires that clinicians engage in active treatment of recurrence and explore biological and clinicopathological characteristics that reflect tumor behavior, such as progressive or metastatic capability [18, 19]. As initially described decades ago, patients with cancer show altered glucose metabolism-related enzyme activity, and the Warburg effect is a key metabolic hallmark of cancer [6]. PKM2 is important for glycolysis in cancers, and has been demonstrated to play a central role in metabolic reprogramming [8, 20, 21]. Recently, using mass spectrometry, Katharina B *et al* demonstrate that there is no evidence for exchange of PKM1 to PKM2 expression during cancer formation [22]. Further study shows that PKM2 isoform switch in cancers is tissue-specific and only occurred in glioblastoma [23]. In addition to functioning as a glycolytic enzyme, PKM2 has also been shown to translocate to the nucleus under certain circumstances. The characterized functions of PKM2 as a transcriptional coactivator and protein kinase endow it with the ability to regulate gene expression [24-28]. In our previous study, we demonstrated that PKM2 has a proliferative advantage and increases the Warburg effect in HCC [17]. In the current study, we found that the expression level of PKM2 was significantly higher in HCC than that in the adjacent benign tissues, suggesting that PKM2 may play an important role in HCC.

Immunohistological staining of different solid tumors with anti-PKM2 antibodies revealed strong and homogeneous PKM2 staining in metastases [29, 30]. In accordance with the immunohistological results, the amount of PKM2 was found to increase in patients’ EDTA-plasma. Several studies reported that PKM2 had high sensitivities in auxiliary diagnosis of melanoma [31], lung cancer [32], cervical cancer [33], gastric and colorectal cancer [12, 14]. These findings indicate the importance of PKM2 in the early detection of cancer, evaluation of anti-cancer effects and prognosis. The present study showed that overexpression of PKM2 was correlated with a high TNM stage and level of vascular invasion in primary and validation cohorts. Both the negative expression of TRIM35 and the positive expression of PKM2 were associated with poor prognosis and aggressive tumor behavior in cases of HCC. Although each gene has a significant effect by itself, the combined alterations in their expressions may compound the impact on the prognosis of HCC.

In recent years, the development of molecular biology has led to the successful exploration and identification of biomarkers. To date, a large number of biomarkers have been proposed for HCC progression and aggressiveness. Regulation of both coding genes and noncoding RNAs in HCC has been suggested to have considerable potential for predicting the diagnosis and prognosis of patients with HCC [34-36]. In this study, we identified PKM2/TRIM35 protein markers for predicting TTR based on protein expression data that was obtained immunohistochemically from samples taken from 236 patients with HCC. Further, we successfully validated the discriminative ability of the PKM2/TRIM35 combination for predicting both TTR and OS in an independent validation set of 205 patients with HCC. Moreover, our analysis of the PKM2/TRIM35 combination as a predictor for early recurrence showed that the sensitivity and specificity were substantially improved. We propose that, by combining our results with the findings of previous reports, the simultaneous analysis of multiple genes can further improve the accuracy of our predictions. However, there are also some limitations to this study. As it was a retrospective study, we were obliged to rely on the completeness of the medical records for our analysis. Further studies with prospectively randomized populations are warranted to confirm these promising results.

In conclusion, in the present study, we demonstrated that PKM2 expression was high and TRIM35 expression was low in tumor samples from patients with in HCC. It is clear from the present study that the positive expression of PKM2 and the negative expression of TRIM35 reflect the aggressiveness and poor prognosis of HCC, making their assessment potentially useful in clinical practice. In addition, a multivariate analysis revealed that PKM2/TRIM35 expression was an independent and significant risk factor for recurrence and survival, suggesting that this combination has prognostic value. In the era of personalized medicine, the identification of patients who are at a high risk of early recurrence may provide clinicians with opportunities for early interventions and improve the outcomes of HCC. The findings of the current study may help to determine optimal treatment strategies.

## METHODS

### Patients and specimens

This study involved four independent cohorts of patients with HCC: cohort 1, which included 129 patients with HCC from Qi Dong Liver Cancer Institute (Jiangsu, PR China); cohort 2 (primary group), which included 236 patients with HCC from Zhongshan Hospital (Shanghai, PR China); and cohort 3 (validation group) and cohort 4, which included 205 and 118 HCC patients from Eastern Hepatobiliary Surgery Hospital (Shanghai, PR China). With the exception of patients from cohort 1, the patients had undergone curative resection of their HCCs, which were prepared on tissue microarray slides. The follow-up procedures and postoperative treatments were based on a uniform guideline and have been described previously [37]. Tumor differentiation was graded using the Edmondson grading system. Clinical staging was performed according to the 6^th^ edition of the AJCC/UICC TNM classification system. Time to recurrence and overall survival were calculated from the date of surgery to the date of the first recurrence and death, respectively. Data were censored at the last follow-up for patients without relapse or upon death. Institutional review board approval was obtained and each patient provided written informed consent.

### Antibodies

anti-PKM2 was purchased from Cell Signaling Technology (Danvers, MA, USA). Anti-TRIM35 was obtained from Santa Cruz Biotechnology (Texas, CA, USA), and anti-GAPDH was obtained from KangChen Bio-tech (Shanghai, PR China).

### Quantitative real-time PCR analysis

Total RNA was extracted from tissues or cells with TRIzol reagent (Invitrogen) according to the manufacturer's instructions. Reverse-transcription PCRs (RT-PCRs) were carried out with the Prime-Script RT Reagent Kit (TaKaRa, Dalian, China). Expression levels of the genes were determined by quantitative real-time PCRs and normalized against an endogenous control β-actin using SYBR Premix Ex Taq (TaKaRa). Data were analyzed using a ΔΔCt approach and expressed as the target gene/β-actin ratio [2^−Δ^Ct(target gene-β-actin)].

### Western blot analysis

Cells were harvested by scraping into an SDS sample buffer containing a cocktail of protease inhibitors (Pierce, Rockford, IL, USA). Similar amounts of proteins were loaded into the gel, separated by SDS-PAGE gel electrophoresis, and transferred to a nitrocellulose membrane (Bio-Rad, Hercules, CA, USA). The membrane was blocked with TBST (0.05% Tween 20 in TBS) containing 5% skim milk and then incubated overnight with the indicated antibodies at 4°C. The membrane was washed three times in TBST and then incubated with an HRP-conjugated secondary antibody (Pierce) (1:2000) for 2 hours at room temperature. The immunocomplexes were detected using enhanced chemiluminescence (Pierce).

### Tissue Microarray and Immunohistochemistry

The tissue microarray was constructed as described previously [38]. Core samples were obtained from representative regions of each tumor based on hematoxylin and eosin staining. Duplicate 1-mm cores were taken from different areas of the same tissue block for each case (intratumoral tissue and peritumoral tissue). Serial sections (4μm thick) were placed on slides coated with 3-aminopropyltriethoxysilane. The immunohistochemistry analysis was carried out as described previously [39]. The primary mAbs used were mouse anti-human TRIM35 (1:15) and rabbit anti-human PKM2 (1:800). The TRIM35 and PKM2 immunostaining intensities were scored semi-quantitatively as follows: 0 for negative and 1 for positive. All samples were anonymously and independently scored by two investigators. In case of disagreement, the slides were reexamined, and a consensus was reached by the observers.

### Statistical Analysis

The experiments were repeated at least three times. The significance level of PKM2 mRNA expression in HCC patients was determined by nonparametric Mann-Whitney U tests.. The chi-squared (χ^2^) test was used to evaluate the association between the PKM2 expression and clinicopathological parameters. The cumulative recurrence and survival rates were determined using the Kaplan-Meier method (log-rank test). The Cox multivariate proportional hazards regression model was used to determine the independent factors that influence survival and recurrence based on the investigated variables. ROC curve analysis was used to determine the efficacy of PKM2 and TRIM35 expression in discriminating early recurrence (less than 3 years) from HCC patients. A P value < 0.05 was considered significant. All statistical analyses were performed using the IBM SPSS Statistics V19 package (Armonk, NY, USA).

## SUPPLEMENTARY MATERIAL TABLES AND FIGURES



## List of Abbreviations

AFP: alpha-fetoprotein
AUC: area under the curve
FFPE: formalin-fixed paraffin-embedded
HBV: hepatitis B virus
HCC: hepatocellular carcinoma
IHC: immunohistochemistry
OS: overall survival
PKM2: pyruvate kinase isoform M2
ROC: receiver-operating characteristic
TCGA: the Cancer Genome Atlas
TRIM35: tripartite motif-containing protein 35
TTR: time to recurrence

## References

[1] Jemal A, Bray F, Center MM, Ferlay J, Ward E, Forman D (2011). Global cancer statistics. CA: a cancer journal for clinicians.

[2] Siegel R, Naishadham D, Jemal A (2013). Cancer statistics, 2013. CA: a cancer journal for clinicians.

[3] El-Serag HB (2011). Hepatocellular carcinoma. The New England journal of medicine.

[4] El-Serag HB, Rudolph KL (2007). Hepatocellular carcinoma: epidemiology and molecular carcinogenesis. Gastroenterology.

[5] Seyfried TN, Shelton LM (2010). Cancer as a metabolic disease. Nutrition & metabolism.

[6] Hsu PP, Sabatini DM (2008). Cancer cell metabolism: Warburg and beyond. Cell.

[7] Spratlin JL, Serkova NJ, Eckhardt SG (2009). Clinical applications of metabolomics in oncology: a review. Clinical cancer research : an official journal of the American Association for Cancer Research.

[8] Christofk HR, Vander Heiden MG, Harris MH, Ramanathan A, Gerszten RE, Wei R, Fleming MD, Schreiber SL, Cantley LC (2008). The M2 splice isoform of pyruvate kinase is important for cancer metabolism and tumour growth. Nature.

[9] Luo W, Semenza GL (2012). Emerging roles of PKM2 in cell metabolism and cancer progression. Trends in endocrinology and metabolism: TEM.

[10] Yang W, Lu Z (2013). Nuclear PKM2 regulates the Warburg effect. Cell cycle.

[11] Peng XC, Gong FM, Zhao YW, Zhou LX, Xie YW, Liao HL, Lin HJ, Li ZY, Tang MH, Tong AP (2011). Comparative proteomic approach identifies PKM2 and cofilin-1 as potential diagnostic, prognostic and therapeutic targets for pulmonary adenocarcinoma. PLoS One.

[12] Kumar Y, Tapuria N, Kirmani N, Davidson BR (2007). Tumour M2-pyruvate kinase: a gastrointestinal cancer marker. European journal of gastroenterology & hepatology.

[13] Landt S, Jeschke S, Koeninger A, Thomas A, Heusner T, Korlach S, Ulm K, Schmidt P, Blohmer JU, Lichtenegger W, Sehouli J, Kuemmel S (2010). Tumor-specific correlation of tumor M2 pyruvate kinase in pre-invasive, invasive and recurrent cervical cancer. Anticancer research.

[14] Haug U, Hundt S, Brenner H (2008). Sensitivity and specificity of faecal tumour M2 pyruvate kinase for detection of colorectal adenomas in a large screening study. British journal of cancer.

[15] Liu AM, Xu Z, Shek FH, Wong KF, Lee NP, Poon RT, Chen J, Luk JM (2014). miR-122 targets pyruvate kinase M2 and affects metabolism of hepatocellular carcinoma. PLoS One.

[16] Jia D, Wei L, Guo W, Zha R, Bao M, Chen Z, Zhao Y, Ge C, Zhao F, Chen T, Yao M, Li J, Wang H, Gu J, He X (2011). Genome-wide copy number analyses identified novel cancer genes in hepatocellular carcinoma. Hepatology.

[17] Chen Z, Wang Z, Guo W, Zhang Z, Zhao F, Zhao Y, Jia D, Ding J, Wang H, Yao M, He X (2014). TRIM35 Interacts with pyruvate kinase isoform M2 to suppress the Warburg effect and tumorigenicity in hepatocellular carcinoma. Oncogene.

[18] Rasool M, Rashid S, Arooj M, Ansari SA, Khan KM, Malik A, Naseer MI, Zahid S, Manan A, Asif M, Razzaq Z, Ashraf S, Qazi MH, Iqbal Z, Gan SH, Kamal MA (2014). New possibilities in hepatocellular carcinoma treatment. Anticancer research.

[19] Bruix J, Gores GJ, Mazzaferro V (2014). Hepatocellular carcinoma: clinical frontiers and perspectives. Gut.

[20] Yang W, Lu Z (2013). Regulation and function of pyruvate kinase M2 in cancer. Cancer Lett.

[21] Bluemlein K, Gluckmann M, Gruning NM, Feichtinger R, Kruger A, Wamelink M, Lehrach H, Tate S, Neureiter D, Kofler B, Ralser M (2012). Pyruvate kinase is a dosage-dependent regulator of cellular amino acid homeostasis. Oncotarget.

[22] Bluemlein K, Gruning NM, Feichtinger RG, Lehrach H, Kofler B, Ralser M (2011). No evidence for a shift in pyruvate kinase PKM1 to PKM2 expression during tumorigenesis. Oncotarget.

[23] Desai S, Ding M, Wang B, Lu Z, Zhao Q, Shaw K, Yung WK, Weinstein JN, Tan M, Yao J (2014). Tissue-specific isoform switch and DNA hypomethylation of the pyruvate kinase PKM gene in human cancers. Oncotarget.

[24] Luo W, Hu H, Chang R, Zhong J, Knabel M, O’Meally R, Cole RN, Pandey A, Semenza GL (2011). Pyruvate kinase M2 is a PHD3-stimulated coactivator for hypoxia-inducible factor 1. Cell.

[25] Yang W, Xia Y, Ji H, Zheng Y, Liang J, Huang W, Gao X, Aldape K, Lu Z (2011). Nuclear PKM2 regulates beta-catenin transactivation upon EGFR activation. Nature.

[26] Gao X, Wang H, Yang JJ, Liu X, Liu ZR (2012). Pyruvate kinase M2 regulates gene transcription by acting as a protein kinase. Mol Cell.

[27] Jiang Y, Li X, Yang W, Hawke DH, Zheng Y, Xia Y, Aldape K, Wei C, Guo F, Chen Y, Lu Z (2014). PKM2 regulates chromosome segregation and mitosis progression of tumor cells. Mol Cell.

[28] Luo W, Semenza GL (2011). Pyruvate kinase M2 regulates glucose metabolism by functioning as a coactivator for hypoxia-inducible factor 1 in cancer cells. Oncotarget.

[29] Brinck U, Eigenbrodt E, Oehmke M, Mazurek S, Fischer G (1994). L- and M2-pyruvate kinase expression in renal cell carcinomas and their metastases. Virchows Archiv : an international journal of pathology.

[30] Schneider J, Neu K, Grimm H, Velcovsky HG, Weisse G, Eigenbrodt E (2002). Tumor M2-pyruvate kinase in lung cancer patients: immunohistochemical detection and disease monitoring. Anticancer research.

[31] Ugurel S, Bell N, Sucker A, Zimpfer A, Rittgen W, Schadendorf D (2005). Tumor type M2 pyruvate kinase (TuM2-PK) as a novel plasma tumor marker in melanoma. International journal of cancer Journal international du cancer.

[32] Papadaki C, Sfakianaki M, Lagoudaki E, Giagkas G, Ioannidis G, Trypaki M, Tsakalaki E, Voutsina A, Koutsopoulos A, Mavroudis D, Georgoulias V, Souglakos J (2014). PKM2 as a biomarker for chemosensitivity to front-line platinum-based chemotherapy in patients with metastatic non-small-cell lung cancer. British journal of cancer.

[33] Kaura B, Bagga R, Patel FD (2004). Evaluation of the Pyruvate Kinase isoenzyme tumor (Tu M2-PK) as a tumor marker for cervical carcinoma. The journal of obstetrics and gynaecology research.

[34] Hoshida Y, Villanueva A, Kobayashi M, Peix J, Chiang DY, Camargo A, Gupta S, Moore J, Wrobel MJ, Lerner J, Reich M, Chan JA, Glickman JN, Ikeda K, Hashimoto M, Watanabe G (2008). Gene expression in fixed tissues and outcome in hepatocellular carcinoma. The New England journal of medicine.

[35] Hou J, Zhou Y, Zheng Y, Fan J, Zhou W, Ng IO, Sun H, Qin L, Qiu S, Lee JM, Lo CM, Man K, Yang Y, Yang Y, Yang Y, Zhang Q (2014). Hepatic RIG-I predicts survival and interferon-alpha therapeutic response in hepatocellular carcinoma. Cancer cell.

[36] Giordano S, Columbano A (2013). MicroRNAs: new tools for diagnosis, prognosis, and therapy in hepatocellular carcinoma?. Hepatology.

[37] Gao Q, Wang XY, Qiu SJ, Yamato I, Sho M, Nakajima Y, Zhou J, Li BZ, Shi YH, Xiao YS, Xu Y, Fan J (2009). Overexpression of PD-L1 significantly associates with tumor aggressiveness and postoperative recurrence in human hepatocellular carcinoma. Clinical cancer research : an official journal of the American Association for Cancer Research.

[38] Zhu XD, Zhang JB, Zhuang PY, Zhu HG, Zhang W, Xiong YQ, Wu WZ, Wang L, Tang ZY, Sun HC (2008). High expression of macrophage colony-stimulating factor in peritumoral liver tissue is associated with poor survival after curative resection of hepatocellular carcinoma. Journal of clinical oncology : official journal of the American Society of Clinical Oncology.

[39] Gao Q, Zhao YJ, Wang XY, Qiu SJ, Shi YH, Sun J, Yi Y, Shi JY, Shi GM, Ding ZB, Xiao YS, Zhao ZH, Zhou J, He XH, Fan J (2012). CXCR6 upregulation contributes to a proinflammatory tumor microenvironment that drives metastasis and poor patient outcomes in hepatocellular carcinoma. Cancer research.

