# Supplementary material for: Co-expression of PKM2 and TRIM35 predicts survival and recurrence in hepatocellular carcinoma
Source: Oncotarget. 2014 Dec 11;6(4):2539–48. doi: 10.18632/oncotarget.2991 (PMC4385869; doi:10.18632/oncotarget.2991)
Supplement: Supplementary file 1 [file oncotarget-06-2539-s001.pdf]

Co-expression of PKM2 and TRIM35 predicts survival and recurrence in hepatocellular carcinoma

Supplementary Material

Supplementary table 1: Clinical Characteristics of HCC Patients

Variable	Cohort 1 (n=129)	Cohort 2 (n=236)	Cohort 3 (n=205)	Cohort 4 (n=118)	p Value
Gender					0.779
Female	17	30	33	18	
Male	102	206	172	100	
Age, years					0.058
≤ 51	67	121	100	75	
> 51	52	115	105	43	
Hepatitis history					P<0.001
No	61	3	47	8	
Yes	68	233	156	109	
α-Fetoprotein (ng/ml)					0.127
≤ 20	34	93	75	54	
> 20	76	143	130	64	
Liver cirrhosis					P<0.001
No	70	28	21	63	
Yes	41	208	184	55	
Tumor size (cm)					P<0.001
≤ 5	33	110	80	77	
> 5	38	126	125	41	
Tumor number					P<0.01
Single	NA	194	162	112	
Multiple	NA	42	43	6	
Vascular invasion					P<0.001

No	77	152	68	88	
Yes	10	84	139	26	
TNM stage					0.015
I and II	NA	187	176	107	
III and IV	NA	49	29	11	

Statistical analyses were done by the Fisher's exact test and Chi-square ($\chi2$) test.

Supplementary table 2: Univariate analyses of factors associated with OS and TTR in validation cohort

Factors	OS Relative risk	(95% CI)	p Value	TTR Relative risk	(95% CI)	p Value
Age, years (>51 vs.≤51)	1.243	0.863-1.790	0.242	1.263	0.873-1.827	0.213
Gender (male vs. female)	0.747	0.452-1.235	0.240	0.647	0.381-1.099	0.108
Hepatitis history (yes vs. no)	1.311	0.829-2.071	0.234	1.310	0.834-2.056	0.241
α-Fetoprotein (ng/ml) (>20 vs. ≤20)	1.194	0.820-1.740	0.355	1.102	0.758-1.602	0.612
Tumor differentiation (poor vs. well)	1.432	0.866-2.368	0.145	1.106	0.689-1.775	0.677
Tumor size (cm) (>5 vs. ≤5)	1.000	0.694-1.440	0.999	0.943	0.653-1.360	0.752
Tumor multiplicity (multiple vs. single)	1.863	1.228-2.827	0.003	1.608	1.028-2.515	0.037
Vascular invasion (yes vs. no)	1.936	1.277-2.936	0.002	1.483	1.006-2.208	0.042
TNM stage (III vs. II vs. I)	1.587	1.195-2.108	0.001	1.507	1.127-2.014	0.006
PKM2 (Pos vs. Neg)	1.728	1.149-2.598	0.009	1.541	1.031-2.304	0.035
TRIM35 (Neg vs. Pos)	0.460	0.312-0.677	< 0.001	0.512	0.340-0.772	0.001
Combination of PKM2 and TRIM35						
Overall	1.617	1.268-2.063	< 0.001	1.495	1.163-1.923	0.002
II vs. I	1.358	0.864-2.133	0.184	1.281	0.826-1.987	0.269
III vs. I	2.580	1.610-4.134	< 0.001	2.246	1.381-3.652	0.001

Univariate analysis was calculated by the Cox proportional hazards regression model. Patients were classified into 4 groups according to the levels of TRIM35 and PKM2: group I, TRIM35 positive and PKM2 negative; group II, positive of both markers; group III, TRIM35 negative and PKM2 positive. TNM, tumor-nodes-metastases; CI, confidential interval.

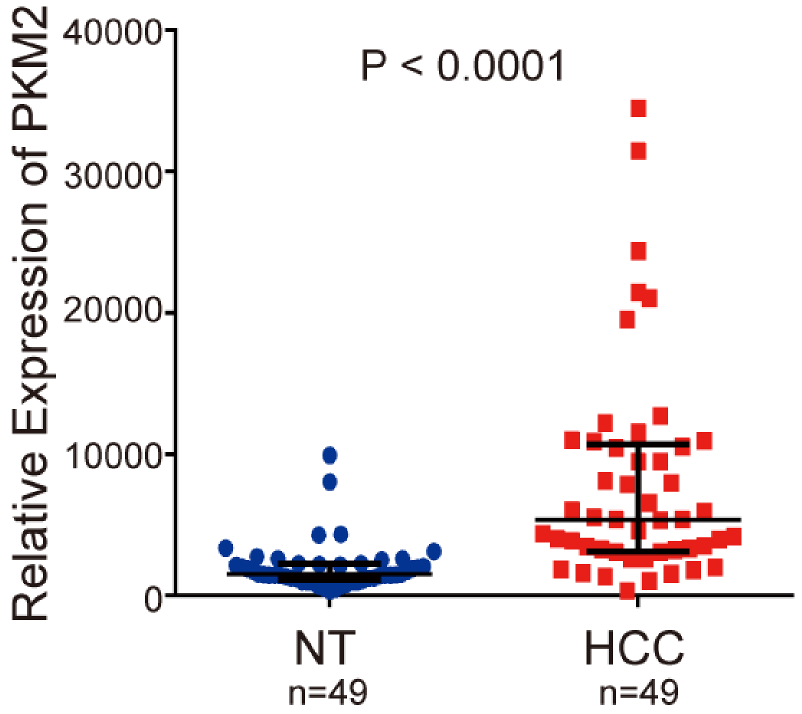

Supplementary figure 1: PKM2 is significantly increased in HCC. The expression levels of PKM2 in 49 HCC and matched adjacent non-tumor liver tissues. Data were depicted as log_2 of the RPKM (reads per kilo bases per million reads).

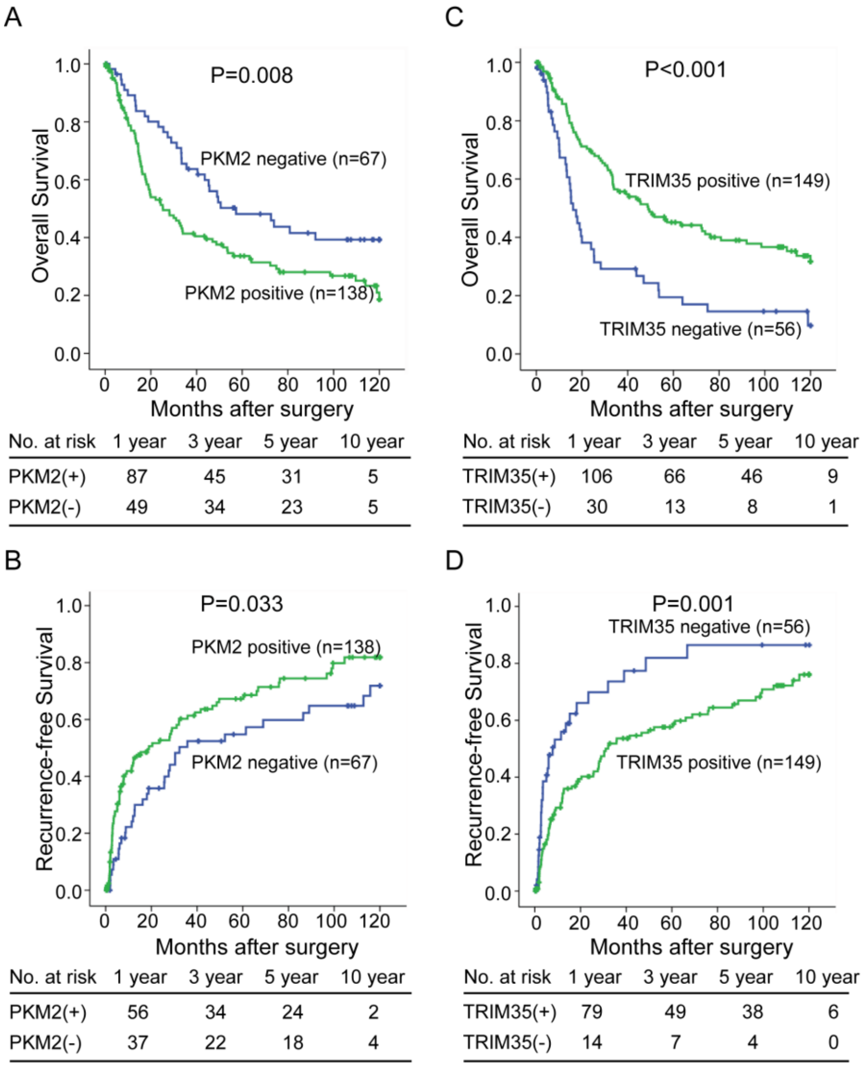

Supplementary figure 2: Positive expression of PKM2 and negative expression of TRIM35 significantly correlates with poor prognosis in HCC patients. (A, B) Kaplan-Meier analysis of the correlation between PKM2 expression and the recurrence-free or overall survival of 205 HCC patients. **(C, D)** Kaplan-Meier analysis of the correlation between TRIM35 expression and the recurrence-free or overall survival of 205 HCC patients. Log-rank tests were used to determine statistical significance.

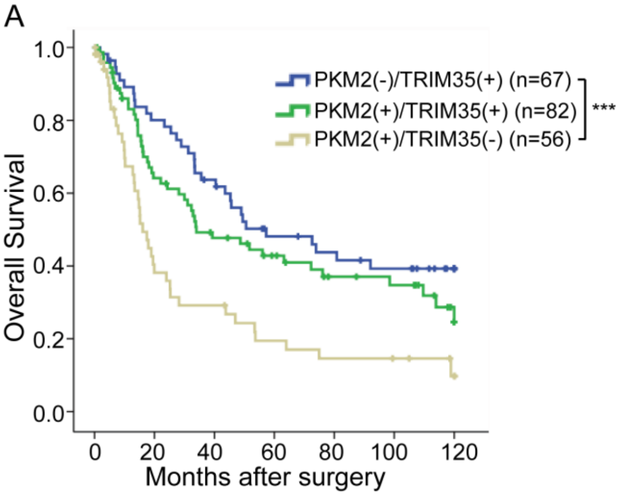

No. at risk	1 year	3 year	5 year	10 year
PKM2(-)/TRIM35(+)	49	34	23	5
PKM2(+)/TRIM35(+)	57	32	23	4
PKM2(+)/TRIM35(-)	30	13	8	1

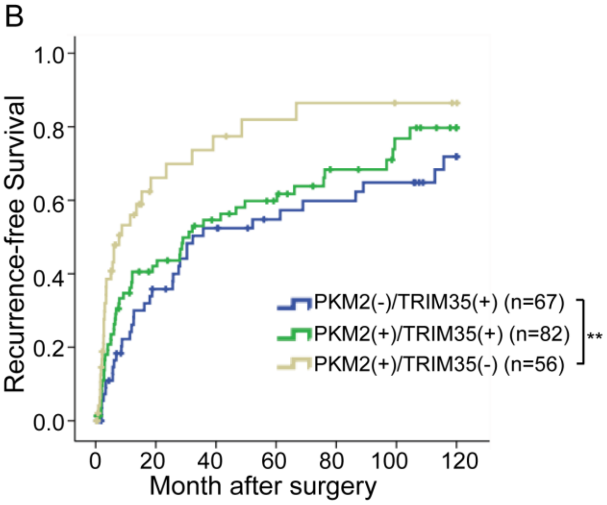

No. at risk	1 year	3 year	5 year	10 year
PKM2(-)/TRIM35(+)	37	22	18	4
PKM2(+)/TRIM35(+)	42	27	20	2
PKM2(+)/TRIM35(-)	14	7	4	0

Supplementary figure 3: Combined influence of PKM2 and TRIM35 dimorphisms on risk of HCC death and recurrence. The association of PKM2/TRIM35 co-expression with recurrence and overall survival in HCC patients. **P < 0.001; ***P < 0.0001.

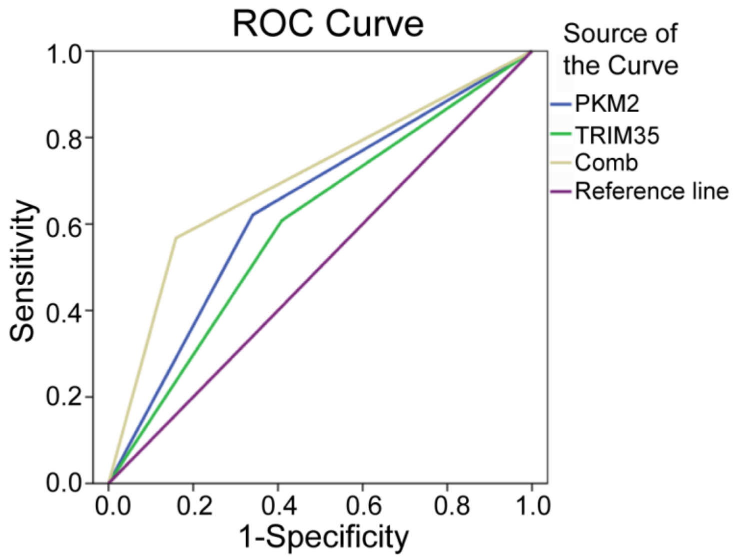

Supplementary figure 4: ROC curve analysis of individual markers and combination of PKM2 and TRIM35 for discriminating early recurrence from HCC patients. AUC was 0.640 for PKM2 (P =0.011), 0.600 for TRIM35 (P =0.07), 0.704 for PKM2 and TRIM35 combinations (P <0.001).
